# Disentangling density and geometry in weather regime dimensions using stochastic twins

**DOI:** 10.1038/s41612-025-01086-w

**Published:** 2025-05-28

**Authors:** Paul Platzer, Bertrand Chapron, Gabriele Messori

**Affiliations:** 1 Laboratoire d’Océanographie Physique et Spatiale, Univ. Brest/Ifremer/CNRS/IRD, Plouzané, F-29280 France; 2 Odyssey, Inria/IMT/CNRS, Plouzané, F-29280 France; 3 Department of Earth Sciences, Uppsala University, Uppsala, 752 36 Sweden; 4 Swedish Centre for Impacts of Climate Extremes (climes), Uppsala University, Uppsala, 752 36 Sweden; 5 Department of Meteorology, Stockholm University, Stockholm, 106 91 Sweden

**Keywords:** Atmospheric dynamics, Statistics

## Abstract

Large-scale atmospheric variability can be summarized by recurring patterns called weather regimes. Their properties, including predictability, have been studied using the local dimension, a geometrical estimate of degrees of freedom from multifractal theory. Local dimension estimates vary across regimes, decrease when a single regime dominates, and increase during transitions, supporting their dynamical significance. However, these variations stem not only from geometry but also from sampling density. We develop a null-hypothesis test using stochastic twins-Gaussian mixture-based surrogates matching atmospheric sampling density but with constant geometry-applied to ERA5 500 hPa fields. Density effects alone explain over 25% of local dimension variance and reproduce the dimension drop near regime peaks, indicating this behavior is density-driven, not geometric. The remaining variability is plausibly geometry-driven. This approach, applicable to any observed system with known sampling distribution, offers a new framework for interpreting local dimension estimates in atmospheric and oceanic data.

## Introduction

Large-scale atmospheric variability can be summarized using recurring patterns called “weather regimes”^1^. They are used as a means of classification of the atmospheric circulation, and each class displays distinct regional weather anomalies that may be used to inform weather forecasts. The North Atlantic and European sector is the region which has seen the most widespread use of weather regimes in the scientific literature. North Atlantic weather regimes correspond to anomalous frequencies of extreme climate events in Europe^2^, anomalies in the ocean circulation^3^, and reflect energy production and demand^4^.

Two representations of weather-regimes coexist in the scientific literature. The first representation focuses on the *recurring* property of weather-regimes, and the latter are therefore seen as statistical objects. In this statistical representation, weather regimes are defined through a fit of the stationary distribution of the data, typically using either k-means clustering^5^ as in^6^ or Gaussian mixture models GMM,^7^ as in^8^. The other representation focuses on the *quasi-stationarity* of the atmospheric circulation around weather regimes, so that the latter are viewed as dynamical objects. In this dynamical representation, the weather regimes are defined as zeros (roots) of the local dynamics^9^ (using a linear approximation). Today, the statistical representation of weather regimes is dominant.

However, the North Atlantic weather regimes reported in the literature mostly overlap in EOF space, so that 1. there is no clear statistical evidence for multimodality^10^, 2. results of clustering algorithms are sensitive to the period considered^11^, 3. using a statistical criterion to determine the optimal number of regimes is challenging^12^. Therefore, one might be concerned about whether these overlapping clusters correspond to physically-meaningful states of the atmospheric circulation.

In the last decade, indicators bridging extreme value theory and dynamical systems theory^13^ have been used to study weather regimes. These indicators are the local dimension (also called instantaneous dimension) and the persistence. They are both derived from an extreme value analysis of analog-to-target distances^14^, and can be computed at each time *t*. It should be stressed that the “extreme values” here are not related to “extreme weather”, but to the distribution of small distances. The persistence relates to the probability for a point in the neighborhood of the actual state to stay in that neighborhood at the next time-step. The local dimension relates to the local geometry of the data, and gives an estimate of the number of dimensions explored by the dataset in the neighborhood around the target point of interest. The local dimension is typically interpreted as an instantaneous measure of predictability^15^. Such indicators have shown that: 1. different weather regimes display differing values of the local dimension and persistence^16^ and 2. local dimension and persistence display an almost-universal behavior of respectively decreasing and increasing around weather regime peaks (*i.e*., the time-passage of the atmospheric circulation through any well-defined regime state)^17–19^. It has been argued that the behavior of these dynamical indicators is a sign of characteristic dynamical features of the atmospheric circulation around regimes. These dynamical indicators may thus be used as informative tools for forecast centers, in conjunction with the classical regime-based analysis. They also support the physical and dynamical relevance of the statistically-derived weather regimes. Indeed, although the clustering classification of weather regimes can be ambiguous, the fact that these cluster-defined regimes bear different dynamical properties from other atmospheric states (higher persistence and lower local dimension) could strengthen confidence in their physical meaningfulness.

Changes in the local geometry can be interpreted as changes in the number of degrees of freedom necessary to describe the local dynamics, and therefore are clearly a dynamical feature of the system. These changes in local geometry originate from the “multifractal” structure of the attractor^20^. For instance, the famous convective “butterfly” attractor of Lorenz^21^ exhibits variations in its local geometry at the crossing of its “wings”: in the wings, the local-geometry is quasi-planar, so that the local dimension is close to 2, while at the crossing of the wings the local dimension increases to ~3 [see supplementary material of ref. ^16^]. Note that in the Lorenz system, all three state-space variables are observed, while we use here only one variable (the 500 hPa geopotential height), so that many degrees of freedom might be missed. However, it was shown that the local dimension applied to observables allows to estimate changes in the projected dynamics^22,23^ also shows on several ideal examples that local dimension estimates are able to capture changes in local geometry. However, local variations in sampling density may also drive variations in estimated local dimensions^24^. Sampling density variations are of a different nature than variations of the local geometry of the data (*i.e*. the multifractal structure). Indeed, changes in sampling density can be observed also for purely random systems with a fixed number of degrees of freedom (*i.e*. constant local geometry). Coming back to the Lorenz attractor,^16^ noted that the local dimension increased at the borders of the wings due to a local decrease of density (see again their supplementary material). However, the local geometry at these locations is unchanged relative to the rest of the wings (quasi-planar). Using simple random variables^25^, showed that there is a negative bias between the true order-1 Renyi dimension^26^ and the sample average of the aforementioned local dimensions, although they should be equal in theory. This bias is stronger for high-dimensional systems and is interpreted as a manifestation of the curse of dimensionality.

**Fig. 1:**
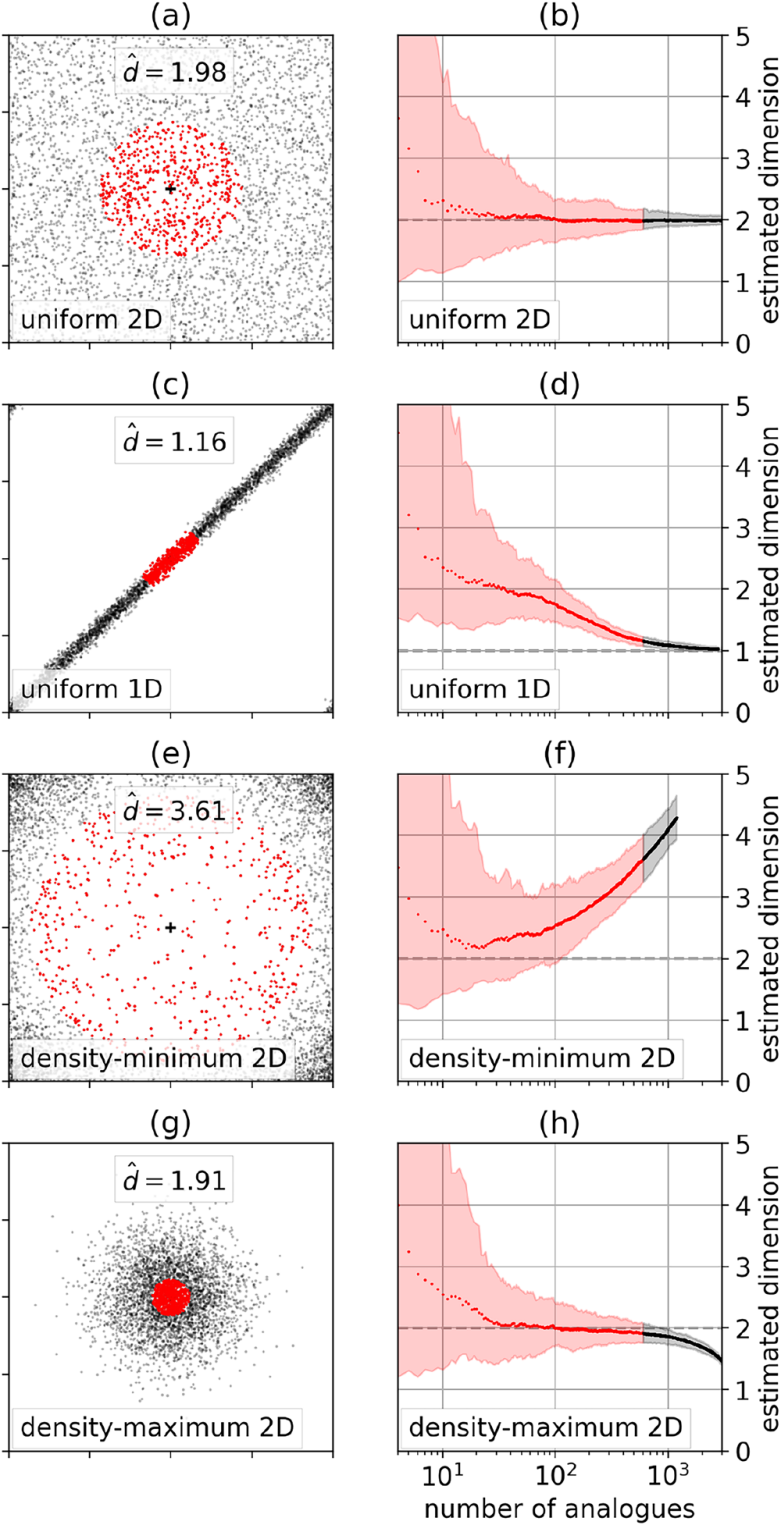
Illustrations of two distinct sources of changes in estimated local dimension: geometry and density. The four distributions are (**a**, **b**) uniform two-dimensional, (**c**, **d**) uniform one-dimensional with small two-dimensional perturbations, (**e**, **f**) two-dimensional local minimum of density, (**g**, **h**) two-dimensional local maximum of density. (a,c,e,g) 4,000 samples from the distribution, with 500 analogs of the target (0,0) in red and the remaining 3500 points in black, and the estimate of local dimension $\hat{d}$ from the red points in text. (b,d,f,h) Median and confidence interval from 2.5% -97.5% percentiles of estimated local dimension $\hat{d}$ for each distribution, as a function of the number of analogs used. Red: with less than 500 analogs. Black: with more than 500 analogs. For each distribution, 100 datasets of 4,000 samples each are generated, allowing to compute the median and percentiles of estimated local dimensions.

Figure 1 illustrates how changes in estimated local dimension can originate from either geometry or density. In the uniform two-dimensional example (Fig. 1a, b), local dimension estimates fluctuate around the expected geometrical value of 2, with increasing confidence with the number of analogs used to estimate the local dimension. In the uniform 1D example (Fig. 1c, d), local dimension estimates approach the expected geometrical value of 1 if the distance to the furthest analog used is significantly larger than the scale of fluctuations around the diagonal line (a similar example was used in^23^ to show that different distances reveal different geometrical features). Thus, the change in local dimension estimates between the uniform 2D and uniform 1D cases is geometry-driven. Conversely, in the density-minimum 2D example (Fig. 1e, f), the estimated dimension does not converge to the expected geometrical value of 2, although the underlying process is inherently two-dimensional. Instead, the estimated local dimension is increasingly biased towards high values as the number of analogs increases. This bias is caused by local changes in density inside the set of analogs (red dots): there are more analogs far away from the target (0,0) than close to it. The opposite effect is observed for the density-maximum 2D example (Fig. 1g, h), with a negative bias that increases with the number of analogs. Thus, the changes in local dimension estimates between the uniform 2D, density-minimum 2D and density-maximum 2D cases are density-driven. This shows that even for a process with constant geometry, local changes in density can influence the value of estimated local dimension, and that this effect is strongest in poorly sampled areas. This illustrates the general result of ref. ^24^.

Based on the above, the present paper addresses the following question: to what extent does the behavior of the local dimension around weather regimes computed through statistical clustering approaches, as observed in refs. ^16–19^, reflect geometry-based versus density-based variations? How can this information be interpreted to understand the physical grounding of weather regimes derived from potentially ambiguous k-means or GMM?

Answering this question demands a dedicated methodology to assess the “density-based” and “geometry-based” sources of local dimension variation. Density-based variations of local dimension are due to uneven sampling of the phase space, and can be observed for purely random variables such as a simple one-dimensional Gaussian variables. Geometry-based variations of local dimension are due to changes in the geometry and can be observed only for particular dynamical systems described by an attractor with a non-smooth invariant measure exhibiting multiple scaling exponents. Density-based variations reflect local changes in the rate of visit of certain parts of the phase space, while geometry-based variations reflect local changes in the number of degrees of freedom of the system. Density-based variations of local dimension do not allow to distinguish random systems from chaotic ones, while geometry-based local dimension variation are a distinctive property of chaotic dynamical systems.

The present work relies on the ERA5 reanalysis^27^ to compute weather regimes based on a GMM of smoothed winter-time 500 hPa geopotential height fields over the North Atlantic ocean and Europe. We use the same tools as^17–19^ to recover typical variations of local dimension around weather regimes. To estimate the contribution of density-based variations, we build “stochastic twins” of the reanalysis in the space of truncated empirical orthogonal functions EOF,^28,29^. These stochastic twins are designed to have a similar sampling density as the reanalysis, although they are absolutely continuous random variables with a constant local geometry (*i.e*. a constant number of degrees of freedom). “Stochastic twins” are related to “surrogate data” used to test a null-hypothesis^30^, typically that the data is generated by a linear process^31^. Instead, here we test for the null-hypothesis that the data has a constant geometry, and therefore we use a different term. We also impose that the stochastic twins have similar time-autocorrelation as the reanalysis, and we generate stochastic twins of the same duration as the reanalysis, allowing to reproduce sampling-based effects. We can then compare, for each target state (*i.e*. each time *t*), the local dimension computed from a neighborhood of points taken from the reanalysis, with the local dimensions computed from neighborhoods of points taken from the stochastic twins. The first is the “full” estimate of local dimension and is thus both density-based and geometry-based, while the second estimate is only density-based. These analyses allow to estimate the fraction of density-based local dimension variations.

The article is structured as follows. The Results section presents the results of the paper, namely that 1. density effects allow to explain at least 25% of the variance of local dimension estimates in general and 2. density effects fully explain decreases in local dimension estimates around weather regimes. The Discussion section recalls the main results and discusses potential future investigations. The Methods section describes the ERA5 dataset, Gaussian-mixture model for weather regimes, weather regime index, and local dimension estimation procedure.

## Results

### Local dimensions over the whole attractor

**Fig. 2:**
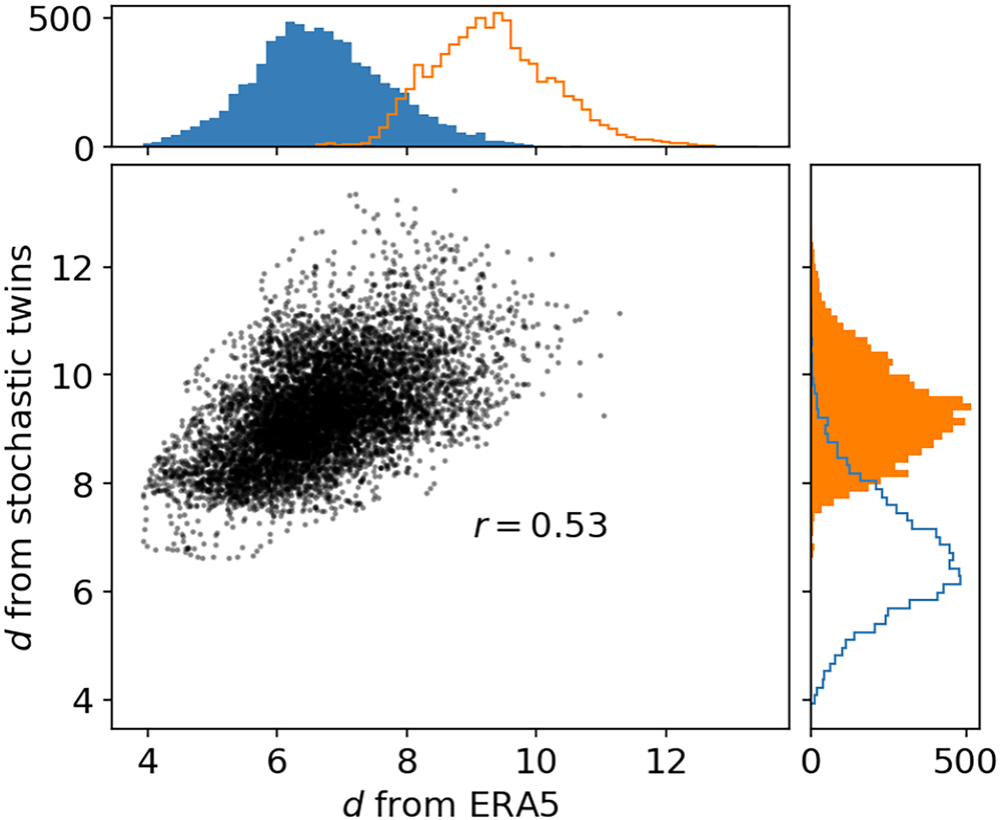
Scatter plot of local attractor dimensions computed using ERA5 data points as neighbours of ERA5 data points in EOF-space (horizontal axis) and using stochastic twin data points as neighbours of ERA5 data points in EOF-space, and averaging over 20 independent twins (vertical axis). Corresponding histograms are shown in full bars, with line histograms replicating the other histogram for comparison. Linear correlation coefficient is indicated.

Figure 2 displays scatter plots of dimension computed using ERA5 data as target states, and either ERA5 or stochastic twins as analogs. To provide stochastic twin estimates, we use one stochastic twin at a time to find analogs and compute then dimension, and then we average the estimated dimensions over all stochastic twins. We use 20 stochastic twins, which appears to be enough to produce stable statistics over the estimated dimensions. When *d* is computed from ERA5, variations of *d* correspond to the “full” local dimension variations, with the combined effects of density and multifractality. On the other hand, when *d* is computed from the stochastic twins, variations of *d* are estimates only of the density-based local dimension variations.

There is a constant offset between the two estimates shown in Fig. 2, with dimension computed from the stochastic twins having an average of ~9 versus ~7 for ERA5, which is expected since random systems tend to fill the available space in all dimensions^32^. The linear correlation coefficient between the two quantities is of ~0.53, so that more than 25% of the variance of local dimensions can be explained by density-induced dimension variations as estimated by the stochastic twins. This indicates that a large fraction of the observed variations of local dimension in atmospheric data could be attributed to local changes in density, independently of changes in local geometry or degrees of freedom. Given the simplicity of our model of stochastic twins, the 0.53 correlation coefficient shown in Fig. 2 should be understood as a lower bound for the true density-induced fraction of local dimension variability. To be more precise: at least 25% of the variability of local dimension estimates in ERA5 reanalysis data *cannot be attributed* to changes in local geometry, since it is reproduced using a system of constant geometry. More sophisticated models might be able to explain a larger fraction of local dimension variations while having a constant number of degrees of freedom. Finally, the correlation coefficient depends on how many analogs *K* are used to compute the local dimensions (Fig. S3 in supplementary information). The correlation coefficient is a strictly growing function of *K*: multifractal features are local, and are therefore hidden when using a larger number of analogs. Also, using a too small number of analogs induces a highly variable estimate of local dimension (see the confidence intervals in Fig. 1b, d, f, h), which decrease correlation between multiple estimators. The fact that the correlation coefficient is still superior to 0.2 even with a low value of *K* = 150 analogs indicates that the density-based local variations are important drivers of estimated dimension changes.

There can be arguments towards computing local dimensions based on the whole fields of smoothed z500 anomaly rather than on projections onto the leading 65 EOFs. This cannot be done when we use the stochastic twins, but we can do it when we compute dimensions based on ERA5 alone. Fig. S4 in the supplementary information replicates Fig. 2, with the difference that the computation of local dimension using ERA5 analogs uses whole fields of z500 anomalies. There is no significant difference between the two figures, showing the robustness of these results.

### Local dimensions of regimes

**Fig. 3:**
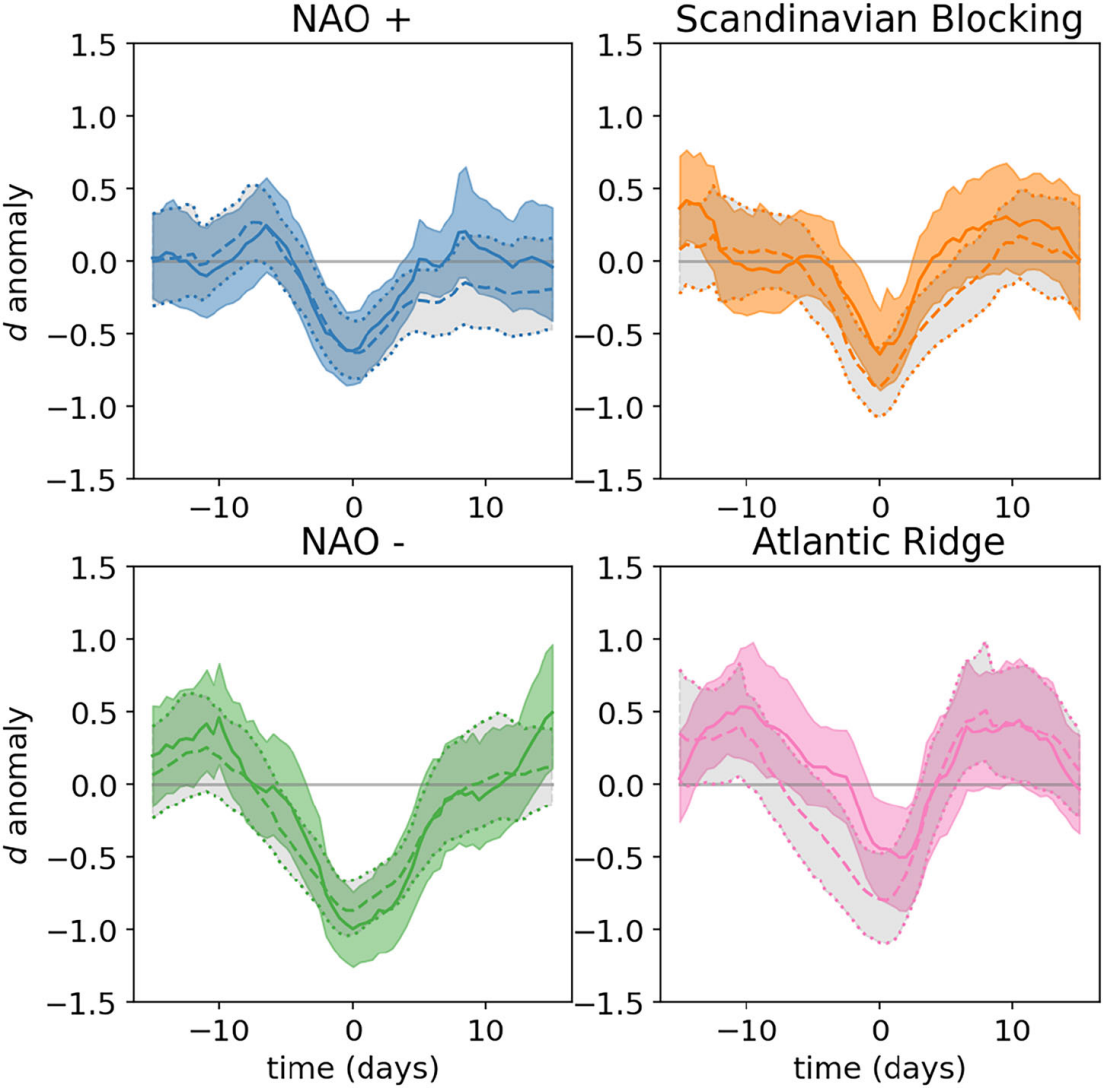
Estimated local dimension anomaly around the time of peak WRI, for each regime. Average over all weather regime life-cycles (lines) and the average’s bootstrap-estimated 95% confidence interval (shades). Full line and color shadings: dimensions computed using ERA5 data points as neighbors (analogs) of ERA5 data points (targets) in EOF-space. Dashed lines and grey color shadings: dimensions computed using stochastic twin data points as neighbors (analogs) of ERA5 data points (targets) in EOF-space, and averaging over 20 independent twins used to computed dimensions.

Next, we investigate whether the dimension estimated from stochastic twins can reproduce the behavior observed in ref. ^17,19^ around weather regimes. Figure 3 shows dimension anomalies around peak WRI in the life-cycles defined in the previous section. The figure shows averages over all life-cycles and bootstrap-estimated 95% confidence intervals for the average. The dimension anomaly is here the difference of local dimension minus a climatological mean computed over ±15 calendar days over the whole available period. For all regimes, there is a significant average decrease of dimension around peak weather regime indices. The agreement between the stochastic-twins-based and natural-data-based estimates of local-dimension falls within the 95% confidence interval for most of the plots. These figures show that the lowered dimension around peak WRI that were observed in ref. ^17,19^ are also found in the stochastic twin-based dimension estimates, and are therefore likely to be mostly density-based rather than geometry-based. This conclusion is coherent with theoretical expressions of density-based variations of local dimension estimates, which predict dimension decreases in an area surrounding the regime centers and extending away from the climatological point of zero-anomaly [see Fig. 4 in ref. ^24^]. Therefore, these dimension decreases largely reflect changes in density which could also be observed for purely random variables with a constant number of degrees of freedom. This implies that the average decrease in local dimension estimate is actually caused by the same subtle changes in density that allow to retrieve weather regimes from statistical clustering algorithms. This conclusion point to the need to qualify the claim that changes in local dimension estimates strengthen evidence for the physical meaningfulness of cluster-defined weather regimes. Namely, this should not be interpreted as a change in the number of degrees of freedom of the atmosphere, but rather as confirmation of the statistical recurrence properties of the regimes.

**Fig. 4:**
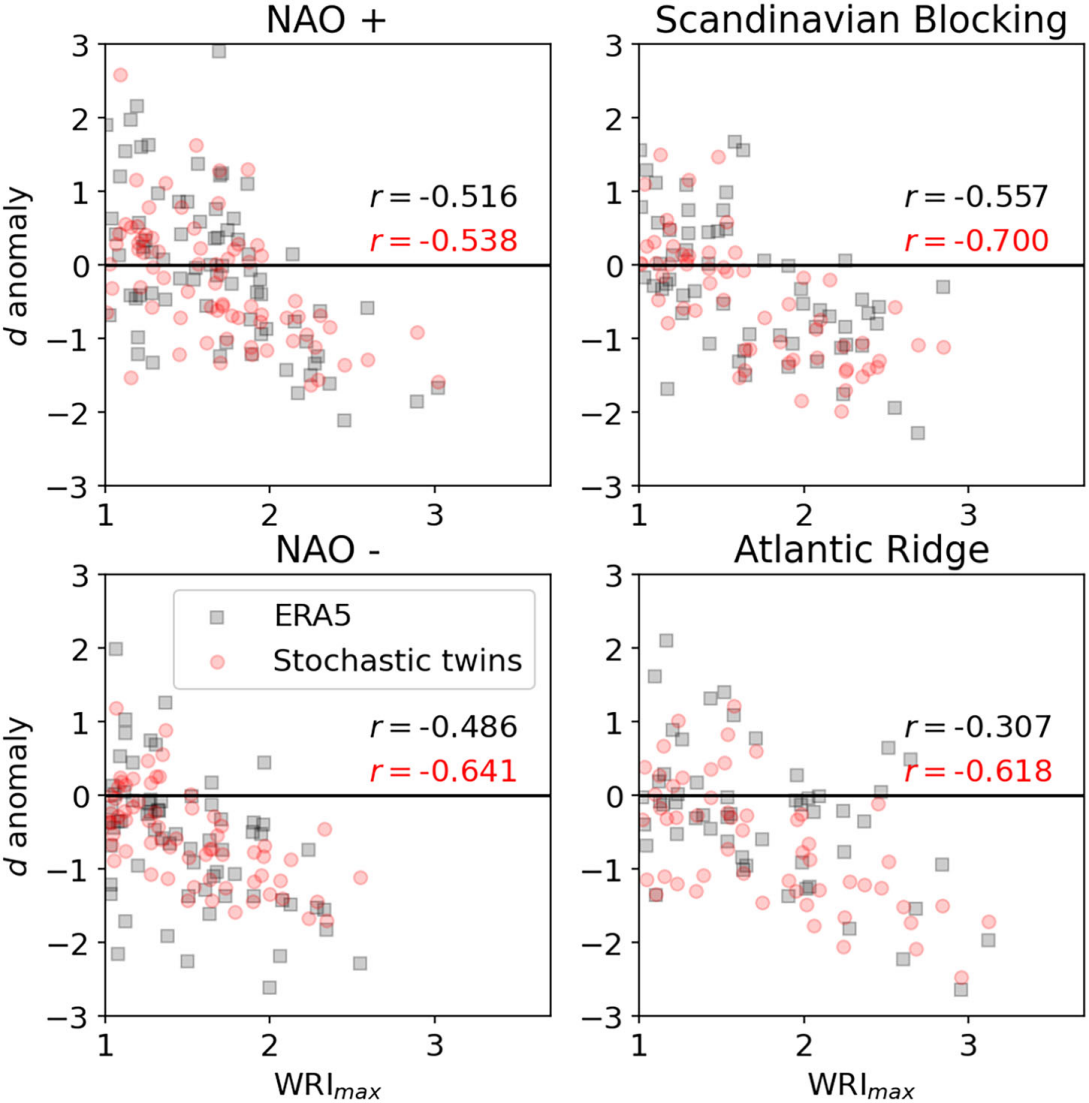
Scatter plot of dimension anomaly at the time of peak WRI, versus peak WRI, for all four regimes. Black squares: dimensions computed using ERA5 data points as neighbors (analogs) of ERA5 data points (targets) in EOF-space. Red circles: dimensions computed using stochastic twin data points as neighbors (analogs) of ERA5 data points (targets) in EOF-space, and averaging over the 20 independent twins used to compute dimensions. Correlation coefficients are shown in black for ERA5 analogs and in red for stochastic twin analogs.

We notice intra-regime variability of the agreement between the stochastic-twins-based and natural-data-based estimates of local dimension. In particular, the onset of the positive phase of the North Atlantic oscillation shows greater agreement than its decay, while the opposite behavior is observed for the Atlantic ridge. Also, for the Scandinavian Blocking and Atlantic Ridge regimes, estimates of local dimension anomalies from stochastic twins are noticeably lower than estimates from natural data alone. Differences between stochastic twin and natural estimates might be the manifestation of actual changes in the local geometry that cannot be reproduced by the geometrically-constant stochastic twins. However, such moderate differences could also be the result of imperfections of our model.

In Fig. S5 of supplementary information, we repeat Fig. 3 but computing local dimension from ERA5 using whole z500 fields, as in Fig. S4. There is no substantial difference except for the slightly stronger dimension anomaly at peak WRI for Atlantic Ridge. However, the dimension anomaly computed from stochastic twin analogs is still stronger than the dimension anomaly computed on ERA5 analogs.

Finally we underscore that the very good temporal agreement between average local dimension anomalies computed with or without stochastic twins in Fig. 3 is due to the fact that we always use ERA5 data points as targets. We can take the analysis one step further by using stochastic twin data alone. To do so, we first compute WRI life-cycles for each stochastic twin as we did for ERA5 projected data. In Fig. S2 in supplementary information, we show the average over all 20 twins of the stochastic twin WRI for each regime life-cycle. This shows an almost-perfectly symmetric behavior of WRI around peak WRI for all regimes, and a more peaked average WRI compared to ERA5 data around the time of peak WRI. The latter is a signature of the erratic, stochastic nature of the stochastic twins. Finally, we compute the average and confidence intervals of local dimension anomaly for each twin over the twin’s regime life-cycles, therefore replicating exactly on each stochastic twin the procedure applied to ERA5 data. Both the life-cycle average and life-cycle confidence intervals are then averaged over the 20 stochastic twins. We also compute the average and standard deviation of dimension anomaly over regime life-cycles, this time computing statistics over all regime life-cycles of the 20 twins. The results of this procedure are shown in Fig. S6 of supplementary information, which can be compared to Fig. 3. In Fig S6, the average dimension anomaly decrease estimated from stochastic twins shows better agreement with that estimated from ERA5 for the NAO+ and Scandinavian Blocking regimes. In Fig. S6, the dimension trough is underestimated (overestimated) from density-based *d* variations compared to full *d* variations for the NAO- (Atlantic Ridge) regime, with stronger differences than in Fig. 3. Finally, in Fig. S6 the confidence interval of averaged dimension anomalies over regime life-cycles is smaller using the stochastic twins rather than ERA5 data as target states. This shows that the average behavior of local dimension around weather regimes is not only a reflection of the average change in local density and geometry at the position of the regimes in phase-space, but also a consequence of the typical trajectories undertaken by the atmospheric circulation around these regimes. Nevertheless, since a similar verdict can be drawn from this second way of computing density-based dimension anomaly around peak WRI, we conclude that the universal behavior of dimension decrease around weather regime life-cycles can largely be attributed to density-based local dimension variations.

To go further, we now try to reproduce the behavior shown in the fourth figure of ref. ^19^. To do so, we plot dimension anomalies at the time of peak WRI against peak WRI. We show both the full dimension anomaly computed from ERA5 analogs of ERA5 targets, and the density-based dimension anomaly computed from stochastic twins analogs of ERA5 targets. For these plots, we only impose a minimum duration of 1 day for the regime life-cycles (versus 8 days for the previous figures), in order to also show smaller peak WRI values and to increase our statistical sample size. Again, for the stochastic-twin-based dimension computation, we take the average over 20 stochastic twins to smooth-out the potential variability associated with the drawing of a single stochastic twin. The result of this procedure is shown in Fig. 4. The negative correlation between *d* anomaly at peak WRI and peak WRI is also witnessed for the density-based *d* anomaly estimated from stochastic twin analogs, and not only for the full local dimension anomaly computed from ERA5 analogs. Again, this shows that the universal behavior of local dimension decrease around regimes is significantly affected by sampling density. Fig. S7 in supplementary information repeats Fig. 4 but with local dimension anomalies from ERA5 analogs computed using whole fields of smoothed z500 anomaly rather than projections on the leading 65 EOFs. This variation does not alter the conclusions drawn from Fig. 4.

Finally, we conduct a similar analysis using stochastic twin data alone, both as targets and as analogs. Following the same procedure as for Fig. S6, we show this time the scatter plots of dimension anomaly at peak WRI against peak WRI for all 20 twins (we therefore have approximately 20 times more points than in the previous scatter plots). The results are shown in Fig. S8 of supplementary information. Again, a negative correlation between dimension anomaly at peak WRI and peak WRI is found, although much weaker for all regimes except for the Atlantic ridge. We attribute this change in behavior to the particular time-trajectories of stochastic twins that do not resemble those of ERA5 around peak WRI. Still, the fact that a strong negative correlation is observed for the Atlantic ridge regime strengthens our claim that the decrease of estimated local dimension around weather regimes can be attributed mostly to density-based effects.

## Discussion

In this paper, we investigate the drivers of changes in the local dimension of weather regimes. We base our analysis on winter-time North Altantic 500 hPa geopotential height fields from the ERA5 reanalysis. This is motivated by two factors. The first is that previous work has used variations of local dimension to interpret the properties of North Atlantic weather regimes and transitions between them. The second is that local dimension estimates can be sensitive to the sampling density of the data they are computed on, as heuristically noted by ref. ^16^ and analyzed in greater detail by ref. ^24^. We specifically distinguish between two possible origins of local dimension variations of the atmospheric circulation: those issuing from changes in multifractal, geometrical properties of the atmospheric flow and those issuing from changes in sampling density. We design a null-hypothesis test to estimate the fraction of density-based variations of local dimension. Our methodology relies on the generation of “stochastic twins” of the system of interest, that bear similar density properties but are purely random with a constant number of degrees of freedom and uniform local geometries. We then estimate the local dimension using analogs drawn from the stochastic twins, and compare the results to local dimension estimates from the ERA5 atmospheric data alone. The local dimension from stochastic twin analogs gives an estimate of the density-based dimension variations, while the local dimension from reanalysis analogs allows to access the full dimension variation, both density-based and geometry-based.

The framework we applied opens for new uses of the local dimension to investigate the chaotic, multifractal properties of the atmospheric circulation and other geophysical phenomena. In particular, one could use the difference between the density-based estimate of dimension anomalies and the full local dimension anomalies, to identify the specific events displaying the largest discrepancies. Here, we have applied our methodology to a single estimator of local dimension. It would be interesting to expand our analysis to other estimators, such as those recently described by ref. ^33^.

Our results suggest that over 25% of the variance of local dimension estimates in the data considered are density-based. The remaining variance is plausibly caused by the irregular geometry of the atmospheric circulation. These estimates do not take into account possible deficiencies of our stochastic twin model. Focusing on weather regimes, we show that the universal behavior of dimension decrease around weather regime peaks is mostly an effect of density. Therefore, the number of degrees of freedom is not necessarily decreasing around weather regime peaks. Our results however only apply to the local dimension. Previous work has characterized weather regimes using both the local dimension and a dynamical systems metric of persistence, which we did not investigate here. Finally, another potential source of variation of local dimension estimates was noted by ref. ^34^, but further investigations are needed to quantify this effect on local dimensions of atmospheric circulation.

We conclude by reformulating the second question posed in the introduction: do variations of the local dimension around weather regimes provide evidence for the physical grounding of regimes ? A positive answer would strengthen our confidence in the regime-based classification of atmospheric patterns, which is debatable from a purely statistical point of view due to the strong overlap between the clusters defining regimes. In light of our results, our answer is “partly”. It appears that the variations in local dimension estimates are primarily caused by the same subtle variations of density that allow for the under-confident clustering of regimes, rather than by changes in geometrical multi-fractal properties. Weather regimes have been widely interpreted in the literature as recurrent and/or quasi-stationary states of the atmospheric circulation. In this respect, variations in the local dimension related to sampling density provide useful information on the regimes, elucidating their recurrent nature. This in turn relates to their physical interpretation as “reference states” of the large-scale atmospheric variability. Nonetheless, conflating density-based and geometry-based variations in local dimension can be problematic. To provide a robust interpretation of the observed changes in local dimension, it is important to understand their origin. This is enabled by the results that we present in this paper.

## Methods

### Generation of illustrative examples

Here we detail how the illustrative examples of Fig. 1 were generated.

For each distribution, each dataset is made of 4,000 points in the square box (−1, 1)^2^. Each random variable is generated in a different way:

1. The uniform 2D in Fig. 1(a,b) is generated from two independent uniform distributions in (− 1, 1) using random.Generator.uniform from the Python package numpy.
2. The uniform 1D in Fig. 1(c,d) is generated as the sum of two random variables:
  A uniform distribution in $(-\sqrt{2},\sqrt{2})$ on the diagonal *x* = *y* line using random.Generator.uniform from the Python package numpy.
  A two-dimensional normal distribution with zero mean and diagonal covariance matrix with both diagonal coefficients equal to 0.25^2^. Each marginal normal distribution therefore has a standard deviation of 0.25.
3. The density-minimum 2D in Fig. 1(e,f) and the density-maximum in Fig. 1(g,h) are both generated from the product of two random variables:
  The cosine $\cos (\Theta )$ of the angle *Θ* with uniform distribution in (0, 2*π*) generated using random.Generator.uniform from the Python package numpy.
  The radius *R* (distance to the origin) with density function proportional to $R\exp (\pm {R}^{2}/{\sigma }^{2})$ for $R < \sqrt{2}$, with positive sign inside the exponential for the density-minimum, and negative for the density-maximum. The factor *R* in front of the exponential ensures that the local density of points is indeed either a decreasing or an increasing function of the distance to the origin. This random variable *R* is sampled using inverse transform sampling, inverting uniform samples in (0, 1) generated using random.Generator.uniform from the Python package numpy. The cumulative distribution function of *R* is approximated numerically using interpolate.interp1d from the Python package scipy.

### Weather regimes from atmospheric reanalysis

This work relies on the ERA5 reanalysis^27^ produced by the European Centre for Medium-Range Weather Forecasts (ECMWF). We use twice-daily (00:00 and 12:00) maps of 500 hPa geopotential height (z500) on a 0.25^∘^-regular grid, for boreal winter (December-January-February, DJF) during the 1979-2023 period (the so-called “satellite era”, when the reanalysis is strongly constrained by remote observations). We focus on the North Atlantic and European sector (30^∘^ < LAT < 88. 5^∘^, -80^∘^ < LON < 40^∘^), similarly to ref. ^6^. Urls and a python code to retrieve the data used in this study are available in the data availability statement.

**Fig. 5:**
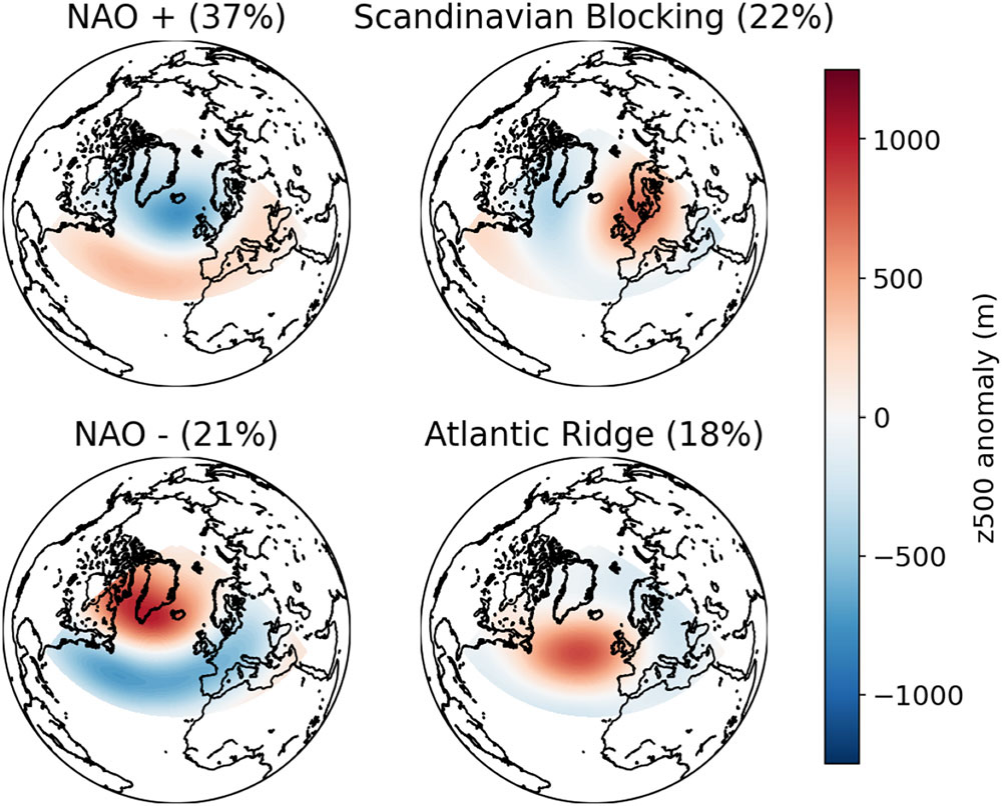
Weather regime means as computed from the best GMM fit using 4 clusters and 3 leading EOFs. Scale is in meters (500 hPa geopotential height). Percentages correspond to the number of days attributed to each regime according to the GMM fit. NAO: North Atlantic Oscillation.

To identify weather regimes, we follow three steps. The first step consists in preprocessing of the gridded data. We first compute anomalies by subtracting a climatology (here a daily climatology, computed as an average over the index “day of year” and on the whole 1979–2023 period). Next, we apply a 10 day low-pass filter (here a simple centered running average). The second step is the decomposition of the covariance of the preprocessed data into empirical orthogonal functions EOF,^28,29^ also called principal component analysis^35^. To do so, we use the python package “eofs.xarray”. The third step is the classification of the data after projection onto a small number of EOFs, using Gaussian Mixture Models GMM,^7^ as in^8^, for which we use here the scikit-learn python package “sklearn.mixture.GaussianMixture”. To do so, we project the smoothed winter-time z500 anomaly fields onto the three first EOFs, normalize by the standard deviation of the first EOF (so that we keep relative variances between EOFs) and perform GMM fit without any constraint on the covariance matrices. We use 3000 different random initializations, and keep only the fit with the best performance in terms of likelihood. The means from this GMM fit as well as the corresponding percentages of days attributed to each regime are shown in Fig. 5.

Although other works usually rely on k-means clustering^5^, we note that the latter can be seen a particular case of GMMs, assuming constant and isotropic covariance matrices for all Gaussian distributions. As our interest is in modeling not only the mean (centers) of the regimes, but the actual distribution, we opt for the GMM which is more flexible. Furthermore, we perform GMM only on the three leading EOF, as it was noted in earlier studies that even when using 12 EOFs for k-means clustering the three first modes actually contribute the most to the position of the regime centers^6^. The choice of performing GMM only on the three leading modes is motivated by the fact that when the covariance matrices of the model are free, the number of parameters scales quadratically with the number of EOFs retained for clustering, increasing the risk of overfitting. We recognize the difficulty of deciding objectively how many components are optimal in a GMM fit^36^, and how many EOFs must be retained for this clustering^8^. We nonetheless underscore that the solutions we retain are relatively stable to the initial condition chosen for the optimization algorithm. Moreover, the resulting regimes perfectly match those described in ref. ^6^ and in ref. ^3^, even though the latter used k-means clustering on 12 EOFs while we use GMM on 3 EOFs.

**Fig. 6:**
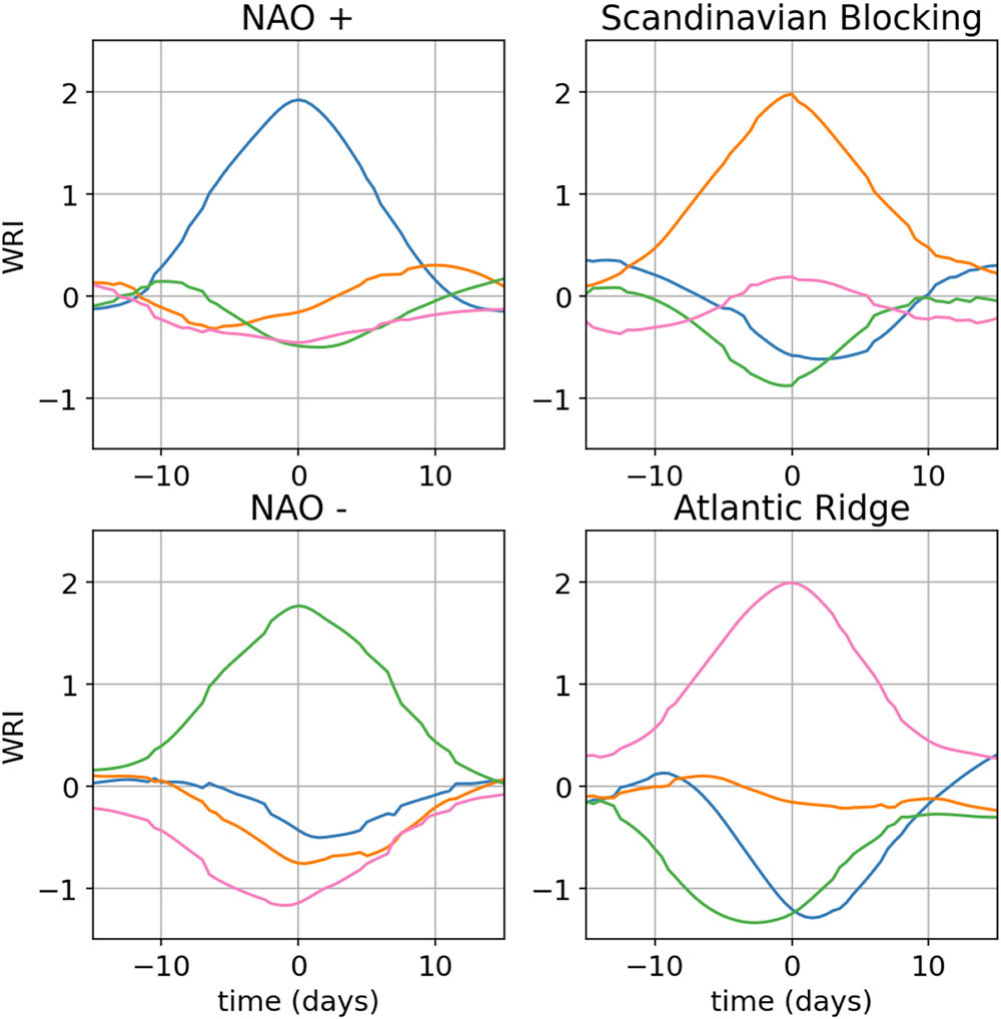
Weather regime index (WRI) averaged around time of maximum WRI, derived from ERA5 data, for life-cycles of at least 8 days during which WRI > 1.

To allow for comparisons with previous literature, we also define a weather-regime index (WRI)^17,19,37^ as the scalar product between the regime center and the projection of ERA5 smoothed z500 anomaly fields onto the three first EOFs. Since we use only three EOFs, our WRI can be reconciled with the one defined using *e.g*. 12 EOFs and k-means by setting the coordinates of the regime centers to 0 for all EOF_4<*i*<12_). Then, the WRI are standardized to have zero mean and unit variance. Finally, weather-regime life-cycles are defined as consecutive days for which WRI_*i*_ > 1. Figure 6 shows average WRI during life-cycles of at least 8 days for all four regimes (8 is a value similar to the 5 days used in ref. ^17^ and the 5-to-15 days used in ref. ^19^). This shows that all four regimes are, on average, well defined and separated during the life-cycles.

### Stochastic twin of atmospheric dynamics

To assess the effect of sampling density on the estimates of local dimension, we use “stochastic twins” of the atmospheric dynamics. These are designed to bear a similar probability density function and time auto-correlation to the ERA5 data. We first use the results of the previous section on GMM fit to generate stochastic twins of the ERA5 data projected onto the three leading EOFs. We use a simple stochastic potential partial differential equation^38^ defined by:

1 $${\rm{d}}{{\bf{x}}}_{123}=-\frac{\partial V}{\partial {{\bf{x}}}_{123}}{\rm{d}}t+{\rm{d}}{\bf{W}}\,,$$

where **x**_123_ is a three dimensional vector, and *V*(**x**_123_) is a three-dimensional potential with gradient $\frac{\partial V}{\partial {\bf{x}}}$, d*t* is the time-increment and d**W** is a Wiener process (a Gaussian random variable of variance d*t*). This simple equation is solved iteratively with an Euler-Maruyama method^38^. Practically speaking, approximate solutions [**x**_123_(0), **x**_123_(1), …, **x**_123_(*L*)] of size *L* + 1 from Eq. (1) are found by drawing a 3*L*-size i.i.d. Gaussian variable of mean zero and variance d*t* to find the values of d**W**, and iterating Eq. (1) from any initial condition **x**_123_(0). For simplicity, we set **x**_123_(0) = (0, 0, 0) for all stochastic twins, which does not influence the results as memory of the initial condition is rapidly lost.

**Fig. 7:**
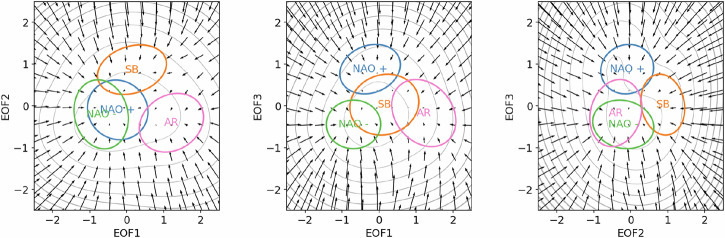
Potential (grey contours) and associated drift (arrows) used in Eq. (1) for the generation of the first three components of the stochastic twins. For each regime, the short names are placed at the regime means. The colored ellipses correspond to isoprobability equal to one-half of the peak probability in the regime centers, as given by each regime’s Gaussian distribution according to the best fit from ERA5 z500 anomalies. NAO: North Atlantic Oscillation. AR: Atlantic Ridge. SB: Scandinavian Blocking.

In order for the solutions of (1) to bear similarities with the projections of the ERA5 smoothed z500 anomaly on the three first EOFs, we set the potential *V*(**x**_123_) so that the static probability density function associated with (1) equals the probability density function of the GMM fit. The latter is given by the GMM fit, and the former is given by the Gibbs measure, thus we solve for:

2 $$\frac{\exp \left(-V({\bf{x}})\right)}{\int\exp \left(-V({\bf{u}})\right){\rm{d}}{\bf{u}}}=\mathop{\sum }\limits_{i=1}^{4}\frac{{\phi }_{i}}{2\det {({{\boldsymbol{\Sigma }}}_{i})}^{1/2}}\exp \left(-{\left({\bf{x}}-{{\boldsymbol{\mu }}}_{i}\right)}^{T}{{\boldsymbol{\Sigma }}}_{i}^{-1}\left({\bf{x}}-{{\boldsymbol{\mu }}}_{i}\right)\right)\,,$$

where we drop the 123-subscript and the values of *ϕ*_*i*_, ***μ***_*i*_ and ***Σ***_*i*_ are given respectively by the weights, averages and covariance matrices from the GMM fit. Therefore the potential is given by:

3 $$V({\bf{x}})=-\log \left\{\sum _{i}\frac{{\phi }_{i}}{2\det {({{\boldsymbol{\Sigma }}}_{i})}^{1/2}}\exp \left(-{\left({\bf{x}}-{{\boldsymbol{\mu }}}_{i}\right)}^{T}{{\boldsymbol{\Sigma }}}_{i}^{-1}\left({\bf{x}}-{{\boldsymbol{\mu }}}_{i}\right)\right)\right\}+{\rm{Cst}}\,,$$

and the constant term has no influence on the resulting Eq. (1). The potential and associated drift are shown along with regime covariance in 3-EOF space in Fig. 7.

Finally, the time-increment is set to d*t* = 0.08 so that the time-auto-correlation of the first component of solutions of Eq. (1) matches that of the first EOF projection of ERA5 twice-daily data, and therefore one time-step in our stochastic twins corresponds to 12 h in atmospheric time-scale.

We now need to have a rule for the generation of other coordinates (>3) of our stochastic twins. Local dimension estimates of the smoothed z500 anomaly fields are of the order of 30 (Fig. S4 in supplementary information shows estimated dimension of fields of smoothed z500 anomaly in the range ~ 20–40). We choose to use 65 coordinates for our stochastic twins. Using 65 EOFs allows to explain 99.91% of the whole variance of smoothed z500 anomaly fields, and adding more coordinates did not change the result of our analysis.

Thus, to define an update rule for the remaining 62 components of our stochastic twins, we assume that each of these higher-order components follows an order-1 auto-regressive process (AR1) :

4 $$\forall i > 3\,,\,{x}_{i}(t+{\rm{d}}t)={\rho }_{i}{x}_{i}(t)+{\varepsilon }_{i}(t)\,,$$

where 0 < *ρ*_*i*_ < 1 is set to match the time-auto-correlation coefficient of the time series of z500 anomalies projected onto EOF_*i*_, and the ${\left\{{\varepsilon }_{i}\right\}}_{i}$ are i.i.d. standard Gaussian random variables. Then, we simply rescale each time series [*x*_*i*_(0), …, *x*_*i*_(*L*)] so that its variance equals that of the z500 anomaly projected onto EOF_*i*_.

**Fig. 8:**
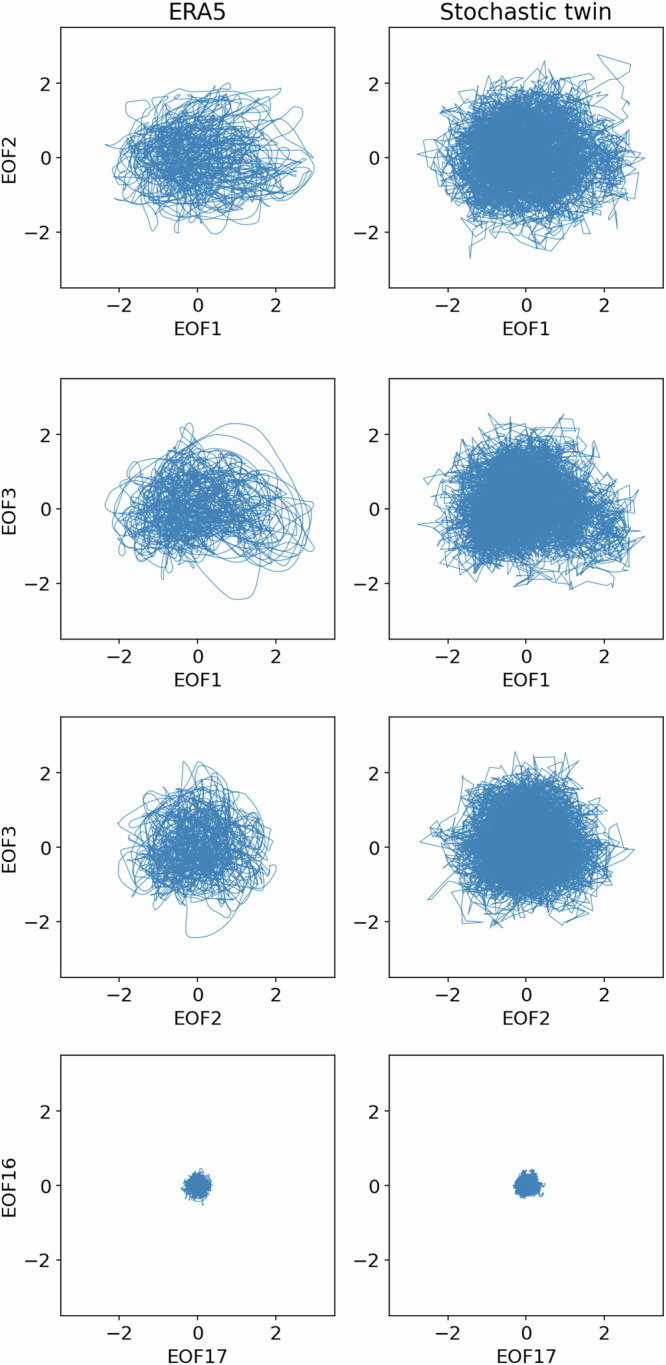
Trajectories in EOF projected space, for coordinates 1vs2, 1vs3, 2vs3, and 16vs17. Left column: ERA5 z500 anomaly, twice-daily. Right column: One stochastic twin.

Figure 8 shows a comparison of the ERA5 z500 smoothed anomaly projected on EOFs 1, 2, 3, 16 and 17 with one stochastic twin of the same duration. The dynamics of the twin show some differences from that of ERA5, notably for EOFs 1, 2 and 3. The stochastic twin has the typical erratic behaviour of potential random systems driven by white noise. On the contrary, ERA5 shows circular-like, smoother trajectories. However, both systems have very similar auto-correlation functions (see supplementary information, Fig. S1), and they share similar probability density functions. Note that the stochastic twin data appear to be denser because they are more erratic and therefore the lines between two consecutive points cover more space on two-dimensional plots. However, the density of points is the same between the ERA5-fitted GMM and the stochastic twins.

### Local dimension analysis

The local dimension is an instantaneous measure of the number of degrees of freedom of the system. It provides information on the geometry of the data in a neighborhood around the point of interest, estimating the number of directions spanned by the neighboring points, also called “analogs”^39^. A high (low) local dimension corresponds to a high (low) complexity of the system, a high (low) number of possible future evolutions of the system, and can be interpreted as a less (more) predictable state.

In this work, we use the same estimator of attractor dimension *d*(**z**) as in previous studies^16,40,41^ :

5 $$d({\bf{z}})={\left\{\frac{1}{K-1}\mathop{\sum }\limits_{k = 1}^{K-1}\log \left(\frac{{r}_{K}}{{r}_{k}}\right)\right\}}^{-1}\,,$$

where *r*_*k*_(**z**) is the Euclidean distance to the *k*-th nearest neighbor or “analog” of **z** in all available data. This estimator is very similar to the one used in ref. ^14^ which is ${\left\{\frac{1}{K}\mathop{\sum }\nolimits_{k = 1}^{K}k\log \left(\frac{{r}_{k+1}}{{r}_{k}}\right)\right\}}^{-1}$. Both are estimators of the scale parameter of the right-tail distribution of the observable ${f}_{{\bf{z}}}:{\bf{x}}\mapsto -\log {\rm{dist}}({\bf{x}},{\bf{z}})$ for any target point **z** in observable space. This distribution is assumed to be exponential, which has been proven to always be the case for a large class of dynamical systems^13^, and also for many systems that fall outside of this class^33^. In this paper, targets **z** and analogs **x** are 65-dimensional vectors, either of EOF-projected z500 smoothed anomaly from ERA5, or from stochastic twins.

Unless otherwise noted, we use a fixed number of neighbors *K* = 450 to compute dimensions for all figure of this article, a number which is coherent with other studies^17,19^. Varying this parameter in the range [150,500] allows for sensitivity tests provided in supplementary information.

## Supplementary information

Supplementary information

## Data Availability

The ERA5 reanalysis is available upon registration at https://cds.climate.copernicus.eu/datasets/reanalysis-era5-pressure-levels?tab=download (last accessed 10th December, 2024). The python code to download the data used in this study is as follows: import cdsapi c = cdsapi.Client() c.retrieve( 'reanalysis-era5-pressure-levels', { 'product_type': 'reanalysis', 'format': 'grib', 'area': [ 88.5, -80, 30, 40, ], 'time': ['00:00','12:00',], 'day': [ '01', '02', '03', '04', '05', '06', '07', '08', '09', '10', '11', '12', '13', '14', '15', '16', '17', '18', '19', '20', '21', '22', '23', '24', '25', '26', '27', '28', '29', '30', '31', ], 'month': [ '01', '02', '03', '04', '05', '06', '07', '08', '09', '10', '11', '12', ], 'year': [ '1979', '1980', '1981', '1982', '1983', '1984', '1985', '1986', '1987', '1988', '1989', '1990', '1991', '1992', '1993', '1994', '1995', '1996', '1997', '1998', '1999', '2000', '2001', '2002', '2003', '2004', '2005', '2006', '2007', '2008', '2009', '2010', '2011', '2012', '2013', '2014', '2015', '2016', '2017', '2018', '2019', '2020', '2021', '2022', '2023', ], 'pressure_level': '500', 'variable': 'geopotential', 'download_format': 'unarchived' }, 'ERA5_2023.grib') # put here your desired file name Note that the preprocessed ERA5 data can be downloaded from the github repository mentioned in the \say{Code availability} statement, so that most of the figures of this article can be reproduced without downloading full ERA5 z500 fields.

## Code availability

The code used to produce the figures of this paper is available at https://github.com/PaulPlatzer/WR_stochastic_twins/.
