# Supplementary material for: Disentangling density and geometry in weather regime dimensions using stochastic twins
Source: NPJ Clim Atmos Sci. 2025 May 28;8(1):203. doi: 10.1038/s41612-025-01086-w (PMC12119342; doi:10.1038/s41612-025-01086-w)
Supplement: Supplementary file 1 — Supplementary information [file 41612_2025_1086_MOESM1_ESM.pdf]

Supplementary Information for *Disentangling Density and Geometry in Weather Regime Dimensions using Stochastic Twins*

Paul Platzer[1,2*], Bertrand Chapron[1,2] and Gabriele Messori[3, 4, 5]

[1]Laboratoire d'Océanographie Physique et Spatiale, Univ. Brest/Ifremer/CNRS/IRD, Plouzané, F-29280, France.
[2]Odyssey, Inria/IMT/CNRS, Plouzané, F-29280, France.
[3]Department of Earth Sciences, Uppsala University, Uppsala, 752 36, Sweden.
[4]Swedish Centre for Impacts of Climate Extremes (climes), Stockholm University, Stockholm, 106 91, Sweden.
[5]Department of Meteorology, Stockholm University, Stockholm, 106 91, Sweden.

*Corresponding author(s). E-mail(s): paul.platzer@ifremer.fr;

This supplementary information is attached to the main paper entitled "Disentangling Density and Geometry in Weather Regime Dimensions using Stochastic Twins".

Fig S1

Fig. S1 compares, on the one hand, the time-autocorrelation functions of the projections of the three leading empirical orthogonal functions of the 10 days running-average of ERA5 500 hPa geopotential height anomaly (full lines), and on the other hand, the three first coordinates of 4 out of the 20 stochastic twins used in this study (dotted lines). This figure confirms that the chosen time-step for our stochastic-twins allows to produces similar auto-correlation decay times as the projected ERA5 data.

Fig S2

Fig. S2 shows the average of weather regime indices (WRI) computed over life-cycles defined as exceedances of the threshold WRI>1 for at least 8 consecutive days and centered on the time of maximum WRI, for each regime index, and averaged over all

20 stochastic twins. This plot highlights that no information on the asymmetry was included in the stochastic twins. For instance, it is known that there are preferred transitions between regimes [1], which results in asymmetry around rescaled time in Fig. 6 of the main paper. Also, the curves are more peaked around $t = 0$ than in Fig. 6 of the main paper, highlighting the erratic nature of the stochastic twins compared to ERA5, also witnessed in Fig. 8 of the main paper.

Fig S3

Fig. S3 shows the dependence of the correlation coefficient between density-based local dimension (from stochastic twin analogs) and full local dimension variations (from ERA5 analogs) to the number of analogs K. This correlation coefficient is the one printed in text in Fig. 2 in the main paper. The fact that the correlation coefficient is a growing function of K is expected since for low values of K the estimation of local dimension is more noisy: estimating a tail parameter from fewer data allows to decrease bias but at the cost of increasing variance. Although there is a strong dependence on K, the fact that the correlation coefficients are always relatively large indicates that a large fraction of variations of local dimension estimates are density-based.

Fig S4, S5, S7

Fig. S4 plots the local dimension estimated from whole fields of smoothed z500 anomaly versus the local dimension computed from stochastic twin analogs of *projected* smoothed z500 anomaly. The results are almost identical to the ones of Fig. 2 of the main paper. Similarly, Fig. S5 and S7 show that the results in terms of local dimensions over weather-regime life cycles and peak index are insensitive to the fact that local dimensions are computed on projected rather than full ERA5 fields.

Fig S6, S8

Fig. S6 and S8 repeat the operations of the main paper but using only stochastic twins (both as targets and analogs). For Fig. S8, all peak WRIs from the 20 stochastic twins are used, which is why the plot is more dense than Fig. 4 of main paper. In the case of Fig. S6 and S8, the results differ significantly from the case where ERA5 data is used as target. In particular, the decrease of estimated local dimension is much weaker when using stochastic data alone for NAO-, while it is stronger in absolute value for Atlantic Ridge. However, the observed effect of decreased dimension around peak WRI is still witnessed using stochastic twin data alone, confirming that density variations play a role in the decrease of estimated local dimension near peak weather regime indices.

References

[1] Kondrashov, D., Ide, K., Ghil, M.: Weather regimes and preferred transition paths in a three-level quasigeostrophic model. Journal of the atmospheric sciences **61**(5), 568–587 (2004)

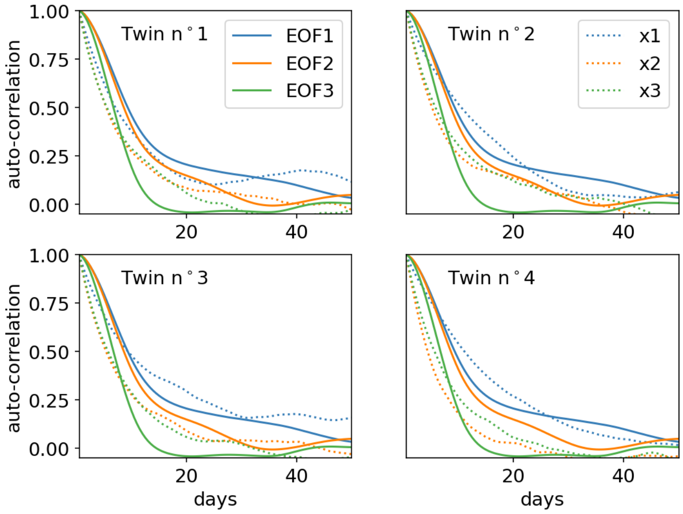

Fig. S1 Auto-correlation functions for EOF_{123}, compared to auto-correlation functions of 4 stochastic twins.

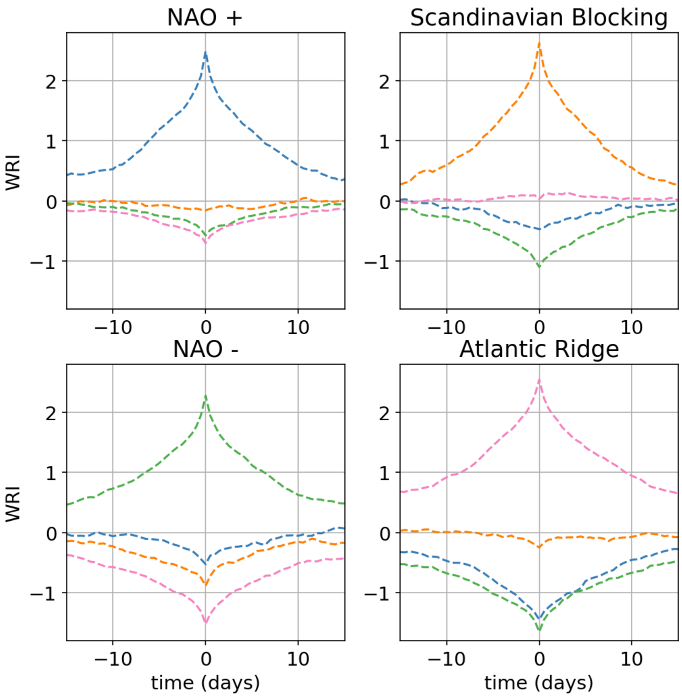

Fig. S2 Weather regime index (WRI) averaged around time of maximum WRI, for life-cycles of at least 8 days during which WRI>1.

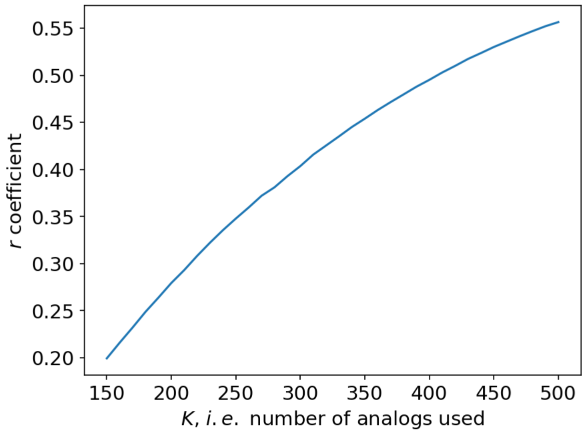

Fig. S3 Sensitivity of the correlation coefficient between density-based local dimension (from stochastic twin analogs) and full local dimension variations (from ERA5 analogs) to the number of analogs K. The coefficient of 0.53 shown in Fig. 2 of main paper corresponds to $K = 450$ analogs.

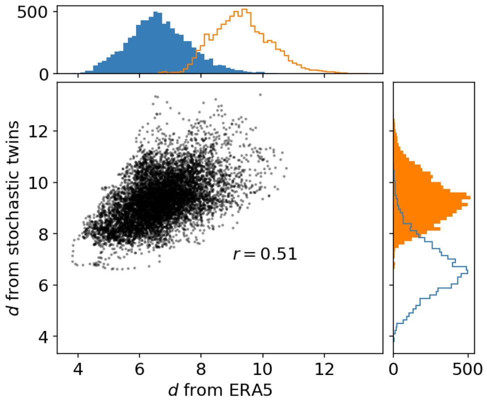

Fig. S4 Same as Fig. 2 of main paper, but the local dimensions from ERA5 alone are computed based on the whole fields of smoothed z500 anomaly rather than on the projection onto the leading 65 EOFs.

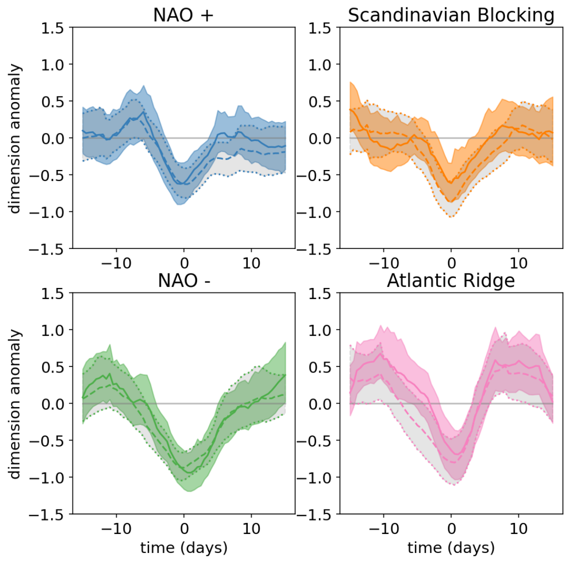

Fig. S5 Same as Fig. 3 of main paper, but the local dimension anomalies from ERA5 analogs are computed based on the whole fields of smoothed z500 anomaly rather than on the projection onto the leading 65 EOFs.

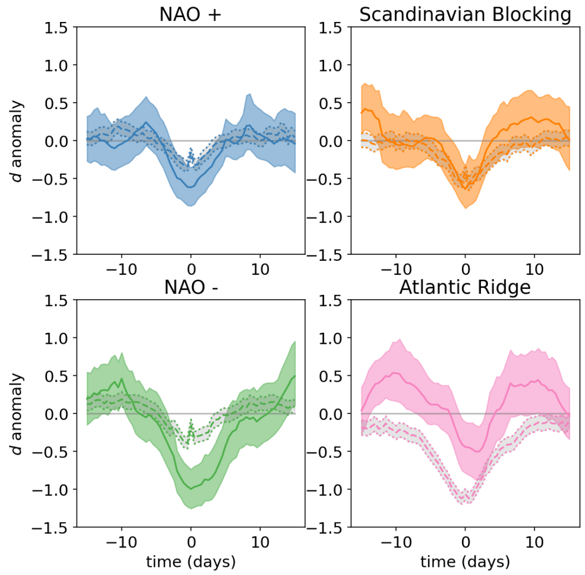

Fig. S6 Same as Fig. 3 of main paper, but here stochastic twins are used both as analogs and as targets to build the dashed curves.

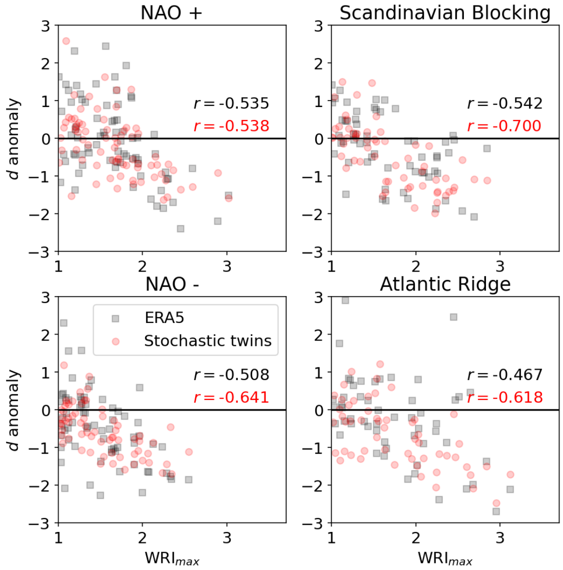

Fig. S7 Same as Fig. 4 of main paper, but the local dimension anomalies from ERA5 analogs are computed based on the whole fields of smoothed z500 anomaly rather than on the projection onto the leading 65 EOFs.

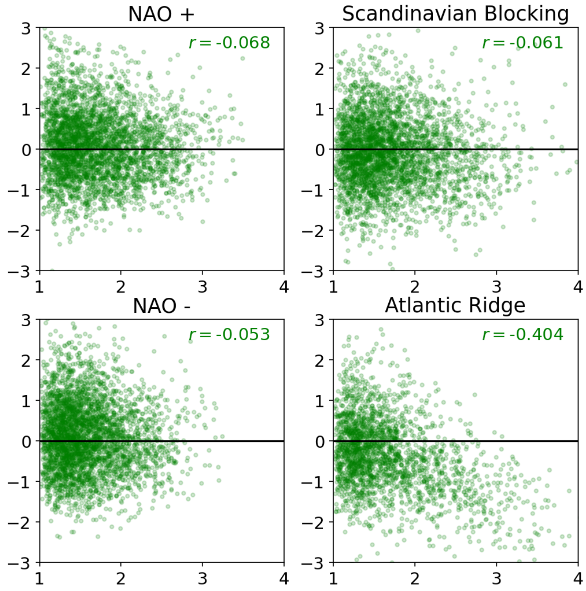

Fig. S8 Same as Fig. 4 of main paper, but using only stochastic twin data.
